# Influence of Sintering Process on Mechanical and Tribological Properties of 3D-Mesh-Structure-Reinforced Cu-Based Friction Materials

**DOI:** 10.3390/ma18235371

**Published:** 2025-11-28

**Authors:** Juxiang Zhu, Yunhai Ma, Zhaoliang Zhang, Lekai Li

**Affiliations:** 1 School of Transportation and Vehicle Engineering, Wuxi University, Wuxi 214000, China; zjx@cwxu.edu.cn; 2 Key Laboratory of Bionic Engineering (Ministry of Education), College of Biological and Agricultural Engineering, Jilin University, Changchun 130022, China; myh@jlu.edu.cn; 3 Weihai Institute for Bionics, Jilin University, Weihai 264200, China; 4 School of Automation, Wuxi University, Wuxi 214000, China; zhangzl@cwxu.edu.cn

**Keywords:** 3D mesh reinforcement structure, sintering process, tribological properties, wear mechanisms

## Abstract

**Background**: Cu-based friction materials (CBFMs) exhibit significant application in transportation and mechanical engineering due to their excellent wear resistance, thermal conductivity, and stable tribological performance. **Methods**: In this study, CBFMs holding a 3D mesh reinforcement structure was prepared under different sintering temperatures and sintering times. The phase, mechanical, and tribological properties were tested, and the wear mechanisms were analyzed. **Results**: The results showed that with an increase in sintering temperature, compressive strength showed a trend of increasing first and then decreasing, COF showed a decreasing trend first and then an increasing trend, and wear rate showed a decreasing trend that can be attributed to the different strength of the matrix and 3D mesh reinforcement structure at different sintering temperatures. With an increase in sintering time, COF continuously increased and wear rate sustained a decrease. **Conclusions**: Compared with previous studies, this study revealed the influence mechanism of sintering temperature and sintering time on the comprehensive properties of CBFMs holding a 3D mesh reinforcement structure for the first time. The results can provide data support for the performance improvement of CBFMs holding a 3D mesh reinforcement structure, and lay a theoretical foundation for the further study of powder metallurgy materials.

## 1. Introduction

Fiction materials, as the critical safety components in transportation equipment such as automobiles, high-speed trains, and construction machinery, directly determine braking efficiency, reliability, and service life [1,2]. Ideal brake friction materials must simultaneously meet multiple performance requirements, including stable friction coefficient (COF), low wear rate, excellent mechanical strength, and high-temperature stability [3,4]. Cu-based friction materials (CBFMs), because of their outstanding thermal conductivity, heat resistance, and mechanical strength, have demonstrated significant application potential in these fields, making them a current research focus [5,6].

Despite the Cu matrix offering high thermal conductivity and high strength, it also exhibits low hardness, which makes it prone to plastic deformation and adhesive wear under high-speed and heavy-load conditions, leading to significant COF fluctuations and an increasing wear rate [7,8], and becoming the critical inherent contradiction of CBFMs. To address this limitation, many reinforcements have been incorporated to enhance the hardness and wear resistance. Nevertheless, conventional randomly distributed reinforcements tend to disrupt the continuity of the Cu matrix, resulting in a notable decline in thermal conductivity and causing stress concentrations at the interfaces. These interfacial stress concentrations can become sites for microcrack initiation, ultimately compromising the overall performance of CBFMs [9,10].

In our previous study, 3D mesh structures constructed by tiny Cu powders could enhance the mechanical and tribological properties to a large extent [11,12]. Due to the continuous spatial framework, controllable pore distribution, efficient stress transfer pathways, and uninterrupted thermal conduction channels, 3D mesh reinforcement structures offer a novel and promising strategy to overcome the limitations of traditional particle-reinforced systems for CBFMs [11]. At the same time, tiny Cu powders enable the construction of dense 3D mesh reinforcement structures through low-temperature sintering and reduce interfacial diffusion resistance between the matrix and reinforcements because of their large specific surface area, high surface energy, and excellent sintering activity [13,14]. Three-dimensional mesh reinforcement structures hold great potential to significantly enhance hardness and wear resistance, and largely preserve superior thermal conductivity and toughness by a sophisticated structural design, thus achieving the synergistic improvement in strength, toughness, and thermal conduction. However, the study on the composites of 3D mesh reinforcement structure/Cu matrix remains in its early stages, especially as the coupled effects of sintering parameters on the microstructure, mechanical, and tribological properties are not yet fully understood. Moreover, the systematic analysis of how wear mechanisms evolve under different processing conditions is still lacking [6,11,15], which significantly hinders the engineering application of such high-performance brake materials.

Although the design concept of 3D mesh reinforcement structures is advanced, the realization of their ultimate performance heavily depends on the critical preparation step of sintering. Sintering parameters such as sintering temperature and sintering time not only directly affect the densification degree and grain size of the Cu matrix, but also determine the interfacial bonding strength, structural integrity of 3D mesh reinforcement structures, and the interactions at the interface between the reinforcement and Cu matrix [11,16,17]. Inappropriate sintering parameters may lead to the collapse of 3D mesh reinforcement structures, abnormal grain growth of the Cu matrix, or weakened interfacial bonding, thus failing to enhance CBFMs. For example, with an increasing sintering temperature from 900 °C to 980 °C, density rose from 6.15 g/cm^3^ to a maximum of 6.45 g/cm^3^, hardness increased from about 45.5 HV to a peak of 62.5 HV, and wear rate decreased dramatically to its minimum of 3.8 × 10^−5^ mm^3^/(N * m) [17]. Existing studies have shown that with an increase in sintering temperature, the atomic diffusion and migration rates accelerate, leading to a reduction in porosity and density, and at the same time, hardness and compressive strength generally exhibited a prior increase trend [18,19]. However, excessively high temperatures will cause grain coarsening and the emergence of sintering defects, which in turn deteriorate the density and hardness [20]. Furthermore, in the wear mechanism analysis, existing reports often attributed wear types to a single factor, overlooking the influence of microstructural differences (such as porosity and interfacial bonding strength) caused by sintering parameters on the transition between wear mechanisms [21,22,23]. Currently, most research has focused on the composition and structural design, while systematic investigations into the effects of sintering parameters on the final performance of 3D-mesh-reinforcement-structure-enhanced CBFMs and the transitions in the wear mechanisms remain insufficient.

To address the aforementioned research gaps, this study employed a tiny Cu powder-based 3D mesh reinforcement structure (constituted by Cu) as the reinforcement to systematically investigate the influence of sintering parameters on the performance of CBFMs (the matrix was Cu-Fe) reinforced by this architecture. The specimen was fabricated at different sintering temperatures and sintering times; the phase, physical/mechanical properties (such as density and compressive strength), and tribological performance (such as COF and wear rate) were thoroughly characterized; and the evolution of dominant wear mechanisms in response to varying sintering processes was analyzed. Furthermore, an intrinsic linkage among sintering process parameters, tribological properties, and wear mechanisms was established. The findings aim to provide a solid theoretical foundation and critical process data to support the optimized fabrication of high-performance CBFMs.

## 2. Materials and Methods

### 2.1. Raw Materials

According to our previous study, the composition of CBFMs in this study is shown in Table 1 [11,24]. The matrix of CBFMs in this paper was a wrap of Cu on Fe; the solid lubricating components were graphite, MoS_2_, and Sb_2_S_3_; the friction components were Al_2_O_3_, SiO_2_, and Cr; and the 3D mesh reinforcement structure was constructed by tiny Cu powders during pressing and sintering according to our previous study [11,12].

### 2.2. Fabrication of CBFMs Holding 3D Mesh Reinforcement Structures

**Figure 1:**
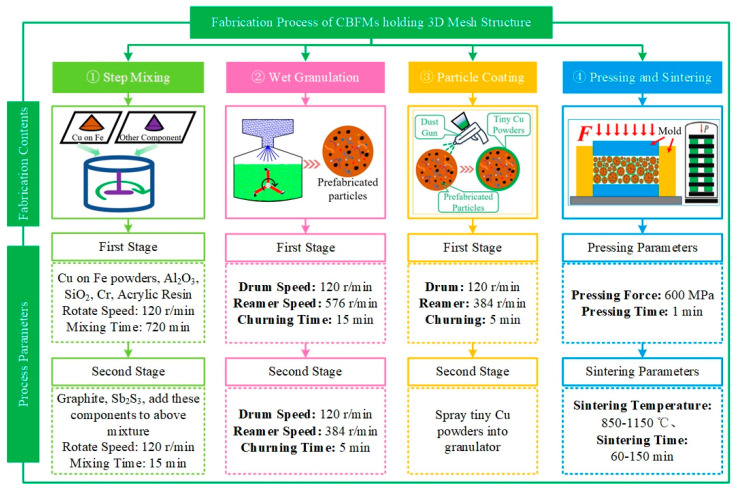
Fabrication process of CBFMs holding 3D mesh reinforcement structures.

The fabrication process of CBFMs holding 3D mesh reinforcement structures in this paper mainly contained step mixing, wet granulation, particle coating, pressing, and sintering (shown in Figure 1).

Raw materials such as the wrap of Cu on Fe, graphite, SiO_2_, and other components were mixed by step mixing using Planetary Ball Milling (QXQM-2, Changsha Tianchuang powder Technology Co., Ltd., Changsha, China) [11,24]. The mixing process is shown in Figure 1.

The mixture of raw materials was prepared into prefabricated particles by wet granulation using a Granulator (JF805R, Jilin Electrical and Mechanical Equipment Co., Ltd, Jilin, China), which can prevent the raw materials powders from gathering. The bridging liquid was Absolute Ethyl Alcohol, and the binder was acrylic resin. The main parameters of wet granulation are shown in Figure 1 [11,24]. After wet granulation, the prefabricated particles were coated with a specific thickness of tiny Cu powders, and the process is shown in Figure 1 [11]. After particle coating, the particles were dried using a vacuum drying oven (JF980S, Jilin Electrical and Mechanical Equipment Co., Ltd, Jilin, China) under 70 °C for 24 h.

The pressing process was conducted in a Hydraulic Machine (LHP-5007, Liaoyuan Steel Back Bearing Co., Ltd., Liaoyuan, China) with a pressure of 600 MPa for 1 min.

**Figure 2:**
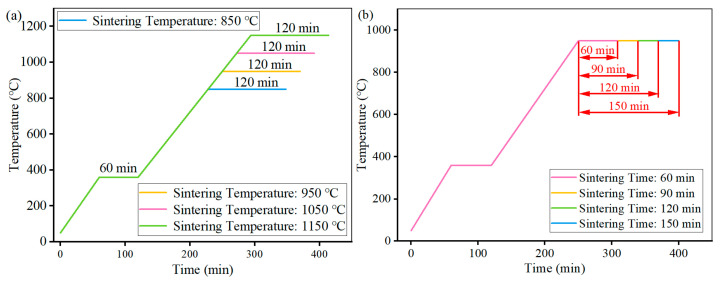
Temperature programming for sintering for (**a**) sintering temperature; (**b**) sintering time.

The sintering parameters are shown in Figure 1 and Figure 2. The sintering device was a Sintering Furnace (69C9, Liaoyuan Steel Back Bearing Co., Ltd., Liaoyuan, China), and the sintering atmosphere was pure hydrogen. Additionally, the purpose of the dwell step below 400 °C is to remove the acrylic resin binder used in the wet granulation and particle coating process.

### 2.3. Phase Analysis and Mechanical and Tribological Properties Test

The phase sample with different sintering parameters was analysis using an X-Ray Diffractometer (XRD, D/max 2500pc, Rigaku, Japan). The scanning range was from 5° to 80°, and the step size was 0.02° [25,26]. The density was obtained by the Archimedes Drainage Method [2,27].

The compressive strength was tested using a Universal Testing Machine (DDL100, Changchun Mechanical Science Research Institute Co., Ltd., Changchun, China). The pressing speed was 0.2 mm/min [24,28]. The geometry of the specimen was cylindrical, whose diameter was 20 mm and height was 7 mm.

The tribological properties were tested using a Friction and Wear Tester (JF75, Jilin Electrical and Mechanical Equipment Co., Changchun, China). The braking speed was 770 r/min, and the braking pressure was 0.4 MPa. COF was calculated according to Equation (1) and wear rate was calculated according to Equation (2) [6,29,30]. To reduce errors, all the samples were tested five times, and the mean value was regarded as the final result.

*μ* = *f*/*F* (1)

where *μ* is the COF; *f* is the friction force (N); and *F* is normal pressure (N).

(2) $$∆T=\frac{1}{2\pi d}\times \frac{A}{n}\times \frac{h_{1}-h_{2}}{f}$$

where *ΔT* is the wear rate (cm^3^ × (N × m)^−1^); *d* is the distance between the brake disc center and specimen center (0.108 m); *A* is the contact surface area (314 mm^2^); *n* is the brake disc revolution during braking; *h*_1_ and *h*_2_ are the thickness of specimen before and after the tribological test, respectively (mm); and *f* is the mean friction force during braking (mm).

After the tribological test, the worn surface micro-morphology and worn surface micro-structure were characterized by a Scanning Electron Microscope (SEM, EVO-18, ZEISS, Jena, Germany) at 20 kV and an Ultra-Depth of Field Microscope (VHX-6000, KEYENCE, Osaka, Japan), respectively.

## 3. Results and Discussion

### 3.1. Phase Analysis

**Figure 3:**
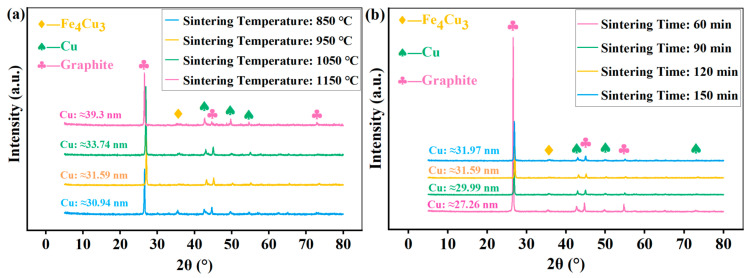
Phase analysis of (**a**) different sintering temperatures and (**b**) different sintering times.

The results of the phase analysis are shown in Figure 3. The phase, grain size, and crystallinity of CBFMs holding a 3D mesh reinforcement structure were different with the difference in sintering temperature (Figure 3a). Specifically, the main phases of all the samples at different sintering temperatures were Cu and graphite. However, an obvious Fe_4_Cu_3_ diffraction peak (about 35.46°) appeared at a sintering temperature of 850 °C, and the diffraction peak of Fe_4_Cu_3_ was weak at a sintering temperature of 1050 °C. Furthermore, at 1050 °C, the diffraction peak intensity of Cu reached the maximum, indicating the maximum crystallization [31]. In addition, it can be seen from Figure 3a that with the increase in sintering temperature (from 850 °C to 1050 °C), the diffraction peak shifted slightly to the right. According to Bragg’s law, this shift towards higher angles indicates a contraction of the crystal lattice. Such lattice contraction is commonly associated with the formation of certain types of lattice defects, such as vacancies or the incorporation of smaller substitutional atoms, which can be promoted by higher sintering temperatures [25].

Figure 3b shows the phase of samples at different sintering times. It can be seen that the sample sintered for 60 min reached the maximum diffraction peak intensity, indicating that a shorter sintering time can lead to a higher crystallinity.

### 3.2. Physical–Mechanical Properties

**Figure 4:**
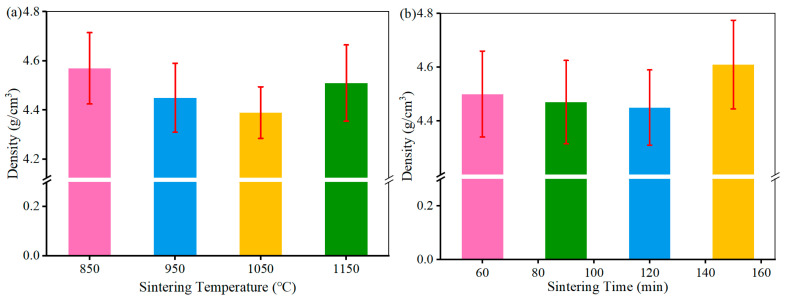
Density of CBFMs fabricated at different (**a**) sintering temperatures and (**b**) sintering times.

The density of CBFMs is of vital significance for its mechanical and tribological properties. Figure 4 shows the density of CBFMs holding 3D mesh reinforcement structures prepared by different sintering processes. It can be seen that with the increase in sintering temperature, density decreased first and then increased. The sample exhibited a de-densification phenomenon as the sintering temperature increased from 850 °C to 1050 °C (which was lower than the melting point of Cu, as shown in Figure 4a). The density of the sample sintered at 1050 °C was 3.94% lower than that sintered at 850 °C. The initial decrease in density could be attributed to the coalescence of pores and the possible expansion of trapped gases during the intermediate stages of solid-state sintering [13].

With a further increase in sintering temperature to 1150 °C (above the melting point of Cu), a liquid phase formed in the sintering system, leading to liquid phase sintering. Interestingly, a slight density reduction was also observed at the initial stage of liquid phase sintering (e.g., at a certain holding time). This transient swelling phenomenon is frequent in liquid-phase sintering when the liquid phase (Cu) wets and rapidly penetrates the grain boundaries of the solid phase. This penetration can temporarily separate solid particles, leading to macroscopic swelling before full densification proceeds via solution-reprecipitation and pore filling [1,2]. However, as the sintering process continued, the enhanced diffusion rate of atoms in the presence of the liquid phase became dominant, promoting material rearrangement and pore elimination. Consequently, the densification degree increased, manifesting as a net increase in density. In addition, with an increasing sintering time, the density showed a trend of decreasing first and then increasing, and the density fluctuation was smaller than that with sintering temperature (shown in Figure 4b).

**Figure 5:**
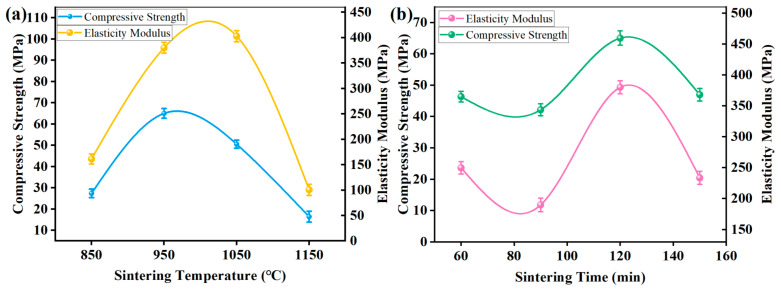
Compressive strength of CBFMs holding 3D mesh reinforcement structure fabricated by different sintering processes of (**a**) sintering temperature and (**b**) sintering time.

Figure 5 shows the compressive strength of the sample prepared by different sintering processes. With an increase in sintering temperature, compressive strength increased first and then decreased (Figure 5a). Sample sintered at 950 °C reached the maximum compressive strength (72.063 MPa), which was 206.89% higher than that sintered at 1150 °C (23.482 MPa). On the one hand, the 3D mesh reinforcement structure constructed by tiny Cu powders would melt and flow into the gap of the surrounding larger particles if the sintering temperature exceeded the melting point (1083 °C), resulting in the disappearance of some or all of the 3D mesh reinforcement structure, thus showing a decreased compressive strength. On the other hand, Cu and Fe in the wrap of Cu on Fe powders were separated at 1150 °C, resulting in the direct contact between iron particles, thus causing decreased bonding and compressive strength. Furthermore, the sample sintered at 950 °C reached the maximum compressive strength, which could be attributed to the optimal 3D mesh reinforcement structure integrity along with strength and interparticle bonding strength. It can be also seen from Figure 5b that the compressive strength showed an increasing trend with the increasing sintering time. In addition, the elastic modulus had the same variation trend as the compressive strength.

### 3.3. Tribological Properties

**Figure 6:**
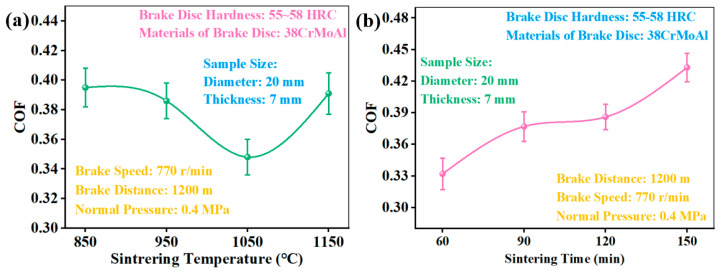
COF of CBFMs holding 3D mesh reinforcement structures fabricated by different (**a**) sintering temperatures and (**b**) sintering times.

The tribological properties of powder metallurgy friction materials largely depend on sintering temperature and sintering time [16,20,32]. Figure 6 showed the COF of CBFMs holding 3D mesh reinforcement structures prepared by different sintering processes. As can be seen from Figure 6a, with an increase in sintering temperature, COF presented a trend of decreasing first and then increasing; COF decreased with the increase in sintering temperature from 850 °C to 1050 °C, which was because of the hinderance by wear debris (caused from the insufficient sintering) [20]. With the increase in sintering temperature, the bonding among powder particles was enhanced, and there was less wear debris at the friction interface, which showed a decreasing COF [33,34]. In addition, with an increasing sintering temperature, the strength of the 3D mesh reinforcement structure constructed by tiny Cu powders also increased. It can act as the nucleation site of wear debris (primary plateaus) during braking, which gathers the wear debris and forms secondary plateaus, thus reducing the COF [11,24]. As the sintering temperature increased to 1150 °C, the Cu was separated from the Fe during sintering (wrap of Cu on Fe powders), and some of the Fe particles leaked onto the friction surface. At the same time, the localized oxide layer at certain spots would fracture, which matched directly with the brake disc, showing an increased COF [13].

Figure 6b shows the COF of CBFMs holding a 3D mesh reinforcement structure prepared at different sintering times. The COF showed an increasing trend with an increasing sintering time. In powder metallurgy, sintering time has a certain effect on comprehensive properties. A longer sintering time can create fuller diffusion among components and higher alloying degrees, thus enhancing the shear strength, manifested as an increasing COF [32].

**Figure 7:**
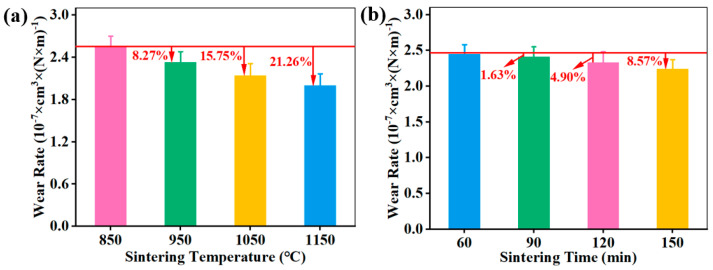
Wear rate of CBFMs holding 3D mesh reinforcement structure by different sintering processes of (**a**) sintering temperature and (**b**) sintering time.

Figure 7 shows the wear rate of CBFMs holding 3D mesh reinforcement structures prepared by different sintering processes. It can be seen that with the increase in sintering temperature, the wear rate showed a gradually decreasing trend. Wear resistance influenced by sintering processes could be attributed to the strength influenced by sintering temperature and sintering time [20,32]. The sintering was insufficient and the bonding among each component was poor at low sintering temperatures. There were more interface defects between the metal matrix and other components, which resulted in the low strength. On the one hand, non-metallic particles (such as SiO_2_, Al_2_O_3_, and graphite, etc.) easily fell off from the matrix, resulting in greater abrasion, and at the same time, the dropped particles formed a third body at the friction interface, which aggravated scratches [13,32]. On the other hand, the strength of the 3D mesh reinforcement structure constructed by tiny Cu powders was also low and could not enhance the strength of CBFMs, thus showing a high wear rate (shown in Figure 7a).

With an increase in sintering temperature, the porosity of the sample gradually decreased, and the internal defects also decreased. The bonding among the raw material particles was enhanced and the materials on the friction surface were difficult to fall off under the shearing stress during braking [13,20,32], thus showing a decreasing wear rate (Figure 7a). With the further increase in sintering temperature (1150 °C), the Cu was in a liquid state. It separated with the Fe, which caused Fe to be exposed on the friction surface, thus enhancing the wear resistance (the wear rate of sample sintered at 1150 °C was 21.26% lower than that sintered at 850 °C).

Figure 7b shows the wear rate of CBFMs holding a 3D mesh reinforcement structure at different sintering times. Too short a sintering time will lead to insufficient sintering, thus influencing the performance of CBFMs; a longer sintering time can improve the performance to a certain extent [13,20]. It can be seen from Figure 7b that the wear rate of CBFMs holding 3D mesh reinforcement structures exhibited a decreasing trend with increasing sintering time. The sample sintered for 150 min reached the minimum wear rate of 2.24 × 10^−7^ cm^3^·(N·m)^−1^, which was 8.57% lower than that sintered for 60 min. Furthermore, by comparing the results in Figure 7a,b, it could be found that the effect of sintering temperature on wear resistance was much larger than that of sintering time.

### 3.4. Wear Mechanisms

**Figure 8:**
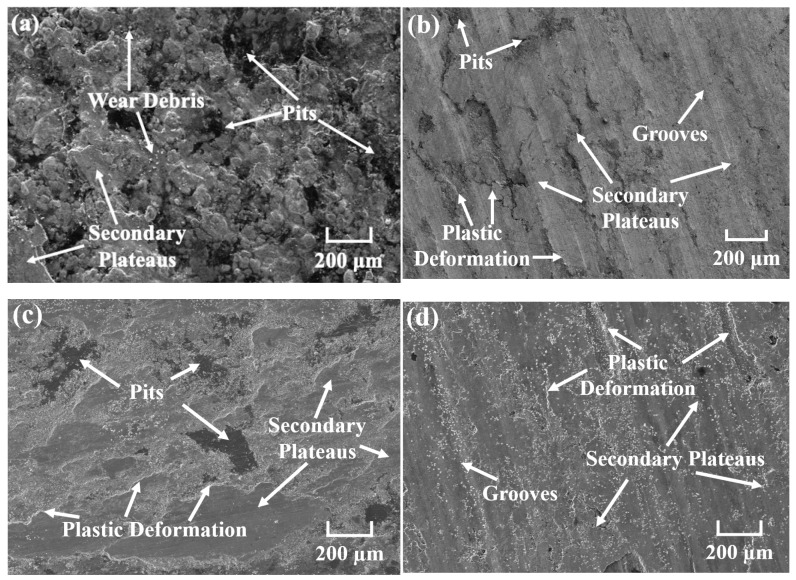
Worn surface micro-morphology at different sintering temperatures of (**a**) 850 °C, (**b**) 950 °C, (**c**) 1050 °C, and (**d**) 1150 °C.

Figure 8 shows the worn surface micro-morphology of CBFMs holding a 3D mesh reinforcement structure at different sintering temperatures. It can be seen that with the increase in sintering temperature, the worn surface became smoother, which corresponded to the gradual decreasing wear rate in Figure 7a. The sintering was insufficient at lower sintering temperatures, and the bonding among raw material particles was poor. During friction, materials on the friction surface would be spalled, resulting in a rough friction surface [11,13]; at the same time, the 3D mesh reinforcement structure constructed by tiny Cu powders could not sinter sufficiently at low temperatures, thus failing to enhance CBFMs and leading to severe wear. As shown in Figure 8a, the worn surface of the sample sintered at 850 °C exhibited numerous large-scale spalling pits and fewer discontinuous secondary plateaus, indicating that the wear mechanism was severe delamination [4,35]. With an increase in sintering temperature (950 °C), the worn surface became smoother, containing large secondary plateaus, numerous plastic deformations, and a small number of discontinuous pits (shown in Figure 8b). The wear mechanism was adhesive wear [1,3]. As the sintering temperature continuously increased (1050 °C), the bonding between raw material particles was significantly strengthened, and the 3D mesh reinforcement structure constructed by tiny Cu powders also reached high strength, thus significantly improving the tribological properties. The worn surface showed slight plastic deformation, a small number of discontinuous spalling pits, and numerous continuous secondary plateaus (shown in Figure 8c). The wear mechanism was slight adhesive wear [27,36]. With a further increase in sintering temperature (1150 °C), Cu remained in a liquid state, and it filled the voids between the larger particles under gravity, which exposed Fe particles on the friction surface. During friction, Fe particles acted as the primary plateaus to promote the formation of large-area secondary plateaus, which can protect the friction materials from being destroyed, thus resulting in the smoothest worn surface (shown in Figure 8d).

**Figure 9:**
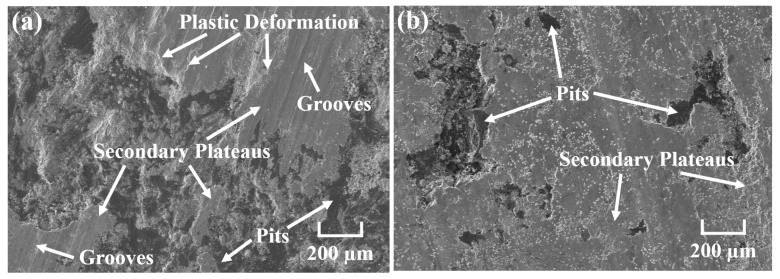

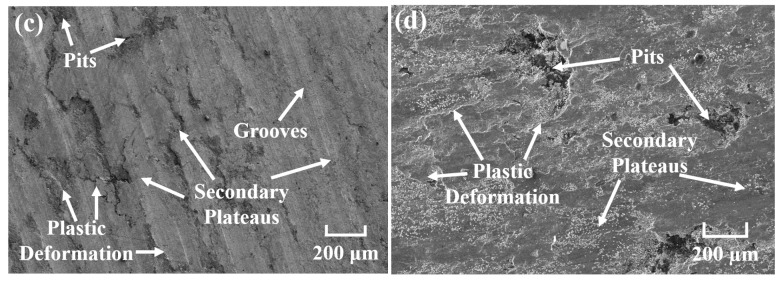
Worn surface micro-morphology at different sintering times of (**a**) 60 min, (**b**) 90 min, (**c**) 120 min, and (**d**) 150 min.

Figure 9 presents the worn surface micro-morphology of CBFMs holding 3D mesh reinforcement structures under different sintering times. It could be observed that the worn surface became smoother with the increasing sintering time. Specifically, the worn surface of the sample sintered for 60 min exhibited discontinuous secondary plateaus, numerous spalling pits and a large number of grooves (shown in Figure 9a), indicating that the wear mechanisms were severe delamination and abrasive wear [37]. As the sintering time increased (90 min and 120 min), the bonding strength between the raw material particles was enhanced, and the strength of the 3D mesh reinforcement structure constructed by tiny Cu powders was also significantly improved. Consequently, the wear resistance of CBFMs was strengthened, manifested by a relatively smooth worn surface with large-area secondary plateaus and a small number of spalling pits (shown in Figure 9b,c). With the further increase in sintering time (150 min), the worn surface featured continuous and dense secondary plateaus, a small amount of plastic deformation, and spalling pits (shown in Figure 9d), corresponding to the minimum wear rate in Figure 7b.

**Figure 10:**
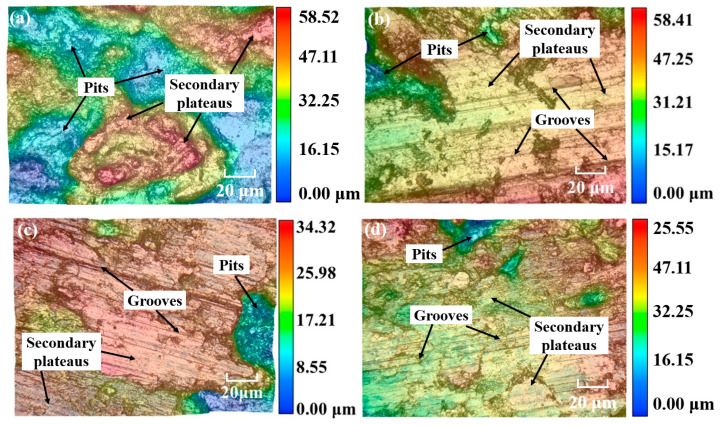
Worn surface micro-structure at different sintering temperatures of (**a**) 850 °C, (**b**) 950 °C, (**c**) 1050 °C, and (**d**) 1150 °C.

Figure 10 shows the worn surface micro-structure of CBFMs holding 3D mesh reinforcement structures with different sintering temperatures. It can be inferred that the worn surface micro-structure was different at different sintering temperatures. As shown in Figure 10a, the strength of the sample sintered at 850 °C was low and there were many defects inside the sample. During friction, the damage occurred first at the defects under shear stress and friction force, and they expanded and connected during the friction process. Finally, a large area of materials was spalled from the friction surface with a depth of up to 58.52 μm, indicating severe wear (which corresponded to the results in Figure 8a). With the increase in sintering temperature, the diffusion rate between matrix particles was accelerated during sintering and the bonding between matrix particles was enhanced, thus improving the strength of the sample. The friction surface was relatively smooth (shown in Figure 10b), and there were small pits with a depth of approximately 58.41 μm on the friction surface (corresponding to the results in Figure 8b). With the further increase in sintering temperature, the strength was further improved, and the friction surface was smoother with smaller and shallower pits (the depth was approximately 34.32 μm). Furthermore, there were also some grooves found on the worn surface in Figure 10c (comparing to Figure 8c), which indicated that there was also slight abrasive wear. The sample sintered at 1150 °C obtained the smoothest worn surface, with only small pits (the depth was about 25.55 μm), presenting the best wear resistance.

**Figure 11:**
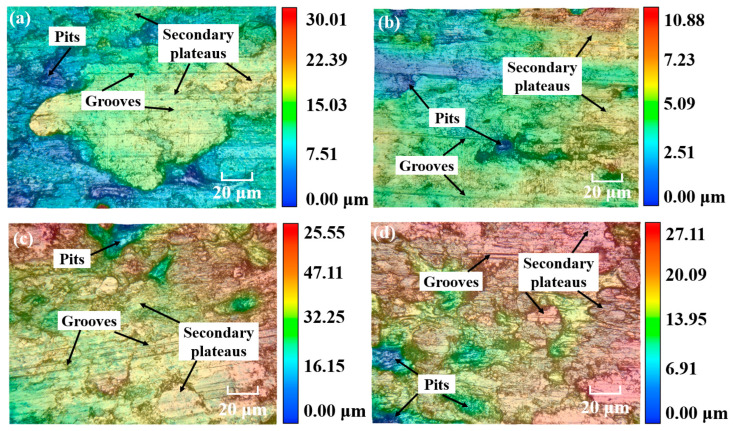
Worn surface micro-structure at different sintering times of (**a**) 60 min, (**b**) 90 min, (**c**) 120 min, and (**d**) 150 min.

Figure 11 shows the worn surface micro-structure of CBFMs holding 3D mesh reinforcement structures with different sintering times. As can be seen in Figure 11, the worn surface was not significantly different for the different sintering times. The worn surface of all the samples had small pits. Among them, the sample sintered for 150 min obtained the smoothest worn surface. Furthermore, compared to the results in Figure 9, it can be inferred that the depth of the pits showed a trend of decreasing first and then increasing with the increase in sintering time.

## 4. Conclusions

This paper investigated the physical–mechanical and tribological properties of CBFMs holding 3D mesh reinforcement structures under different sintering processes. Specifically, single-factor experiments were designed to fabricate specimens under different sintering times and sintering temperatures. The density, phase, physical–mechanical, and tribological properties were tested, and the micro-morphologies and micro-structures of the worn surface were observed to reveal the wear mechanisms. The main conclusions are as follows:

(1) Overall, the influence of sintering temperature on the physical–mechanical properties (such as density and compressive strength) and tribological properties (such as COF and wear rate) of CBFMs holding 3D mesh reinforcement structures was greater than that of sintering time.
(2) Sintering temperature and sintering time exhibited different regulation patterns on the compressive strength of CBFMs holding 3D mesh reinforcement structures. The compressive strength increased first and then decreased with the increase in sintering temperature. The sample sintered at 1150 °C obtained the minimum compressive strength, and the sample sintered at 950 °C obtained the maximum compressive strength. Moreover, sample sintered at 120 min showed the best compressive strength.
(3) Sintering temperature and sintering time had a significant influence on the tribological properties of CBFMs holding 3D mesh reinforcement structures. Specifically, as the sintering temperature increased, COF increased first, then decreased, and finally increased. The sample obtained the minimum COF of 0.348 at 1050 °C. With the increase in sintering time, the COF showed an increasing trend.
(4) Increasing sintering temperature can enhance the wear resistance of CBFMs holding 3D mesh reinforcement structures, and this could mainly be attributed to the improved bonding strength among matrix particles and the increased strength of the 3D mesh reinforcement structure.

This study does not explore the quantitative influence of initial porosity, grain morphology, or other microstructural features on the wear behavior of CBFMs reinforced by 3D mesh structures under different sintering processes. In the future, we will prioritize the systematic investigation of the microstructure (including pore shape, grain size, etc.) under different sintering processes and establish a quantitative relationship with the wear performance, and establish a quantitative structure–property–wear mechanism model by introducing parameters such as resistance to plastic deformation.

## Figures and Tables

**Table 1:** Components for CBFMs.

Components	Content (ωt %)	Powder Size (Mesh)	Manufacturer
Wrap of Cu on Fe	61.5	300	Henan Taihe Huijin Powder Technology Co., Ltd., Jiaozuo, China
Graphite	11	100	Shanghai Youmo Composite Materials Co., Ltd., Shanghai, China
MoS_2_	5	200	Nangong Chunxu Metal Material Factory, Xingtai, China
Sb_2_S_3_	5	200	Zhongke Yanuo (Beijing) Technology Co., Ltd., Beijing, China
Al_2_O_3_	3	100	Suiye Electronic Applied Materials Co., Ltd., Dongguan, China
SiO_2_	0.8	200	Hebei Keze Metal Materials Co., Ltd., Handan, China
Cr	3.7	500	Nangong Xindun Alloy Electrode Spray Co., Ltd., Xingtai, China
Tiny Cu powders	10	2000	Nangong Xindun Alloy Electrode Spray Co., Ltd., Xingtai, China

## Data Availability

The original contributions presented in this study are included in the article. Further inquiries can be directed to the corresponding author.

## Abbreviations

The following abbreviations are used in this manuscript:

CBFMs	Cu-Based Friction Materials
COF	Friction Coefficient
SEM	Scanning Electron Microscope
